# Aadh2p: an *Arxula adeninivorans* alcohol dehydrogenase involved in the first step of the 1-butanol degradation pathway

**DOI:** 10.1186/s12934-016-0573-9

**Published:** 2016-10-12

**Authors:** Marion Rauter, Jakub Kasprzak, Karin Becker, Jan Riechen, Sebastian Worch, Anja Hartmann, Martin Mascher, Uwe Scholz, Kim Baronian, Rüdiger Bode, Frieder Schauer, H. Matthias Vorbrodt, Gotthard Kunze

**Affiliations:** 1 Orgentis Chemicals GmbH, Bahnhofstr. 3-5, 06466 Gatersleben, Germany; 2 Leibniz Institute of Plant Genetics and Crop Plant Research (IPK), Corrensstr. 3, 06466 Gatersleben, Saxony-Anhalt Germany; 3 Jäckering Mühlen-und Nährmittelwerke GmbH, Vorsterhauser Weg 46, 59007 Hamm, Germany; 4 School of Biological Sciences, University of Canterbury, Private Bag 4800, Christchurch, New Zealand; 5 Institute of Microbiology, University of Greifswald, Jahnstr. 15, 17487 Greifswald, Germany

**Keywords:** Alcohol dehydrogenase, 1-butanol, Aadh2p, *Arxula adeninivorans*

## Abstract

**Background:**

The non-conventional yeast *Arxula adeninivorans* uses 1-butanol as a carbon source and has recently attracted attention as a promising organism for 1-butanol production. Alcohol dehydrogenases (adhp) are important catalysts in 1-butanol metabolism, but only Aadh1p from *Arxula* has been characterized. This enzyme is involved in ethanol synthesis but has a low impact on 1-butanol degradation.

**Results:**

In this study, we identified and characterized a second adhp from *A. adeninivorans* (Aadh2p). Compared to *Saccharomyces cerevisiae* ADHs’ (ScAdh) protein sequences it originates from the same ancestral node as ScAdh6p, 7p and 4p. It is also localized in the cytoplasm and uses NAD(H) as cofactor. The enzyme has its highest activity with medium chain-length alcohols and maximum activity with 1-butanol with the catalytic efficiency of the purified enzyme being 42 and 43,000 times higher than with ethanol and acetaldehyde, respectively. *Arxula adeninivorans* strain G1212/YRC102-AADH2, which expresses the *AADH2* gene under the control of the strong constitutive *TEF1* promoter was constructed. It achieved an ADH activity of up to 8000 U/L and 500 U/g dry cell weight (dcw) which is in contrast to the control strain G1212/YRC102 which had an ADH activity of up to 1400 U/L and 200 U/g dcw. Gene expression analysis showed that *AADH2* derepression or induction using non-fermentable carbon-sources such as ethanol, pyruvate, glycerol or 1-butanol did occur. Compared to G1212/YRC102 *AADH2* knock-out strain had a slower growth rate and lower 1-butanol consumption if 1-butanol was used as sole carbon source and AADH2-transformants did not grow at all in the same conditions. However, addition of the branched-chain amino acids leucine, isoleucine and valine allowed the transformants to use 1-butanol as carbon source. The addition of these amino acids to the control strain and Δ*aadh2* mutant cultures had the effect of accelerating 1-butanol consumption.

**Conclusions:**

Our results confirm that Aadh2p plays a major role in *A. adeninivorans* 1-butanol metabolism. It is upregulated by up to 60-fold when the cells grow on 1-butanol, whereas only minor changes were found in the relative expression level for Aadh1p. Thus the constitutive overexpression of the *AADH2* gene could be useful in the production of 1-butanol by *A. adeninivorans,* although it is likely that other *ADHs* will have to be knocked-out to prevent 1-butanol oxidation.

**Electronic supplementary material:**

The online version of this article (doi:10.1186/s12934-016-0573-9) contains supplementary material, which is available to authorized users.

## Background

**Fig. 1:**
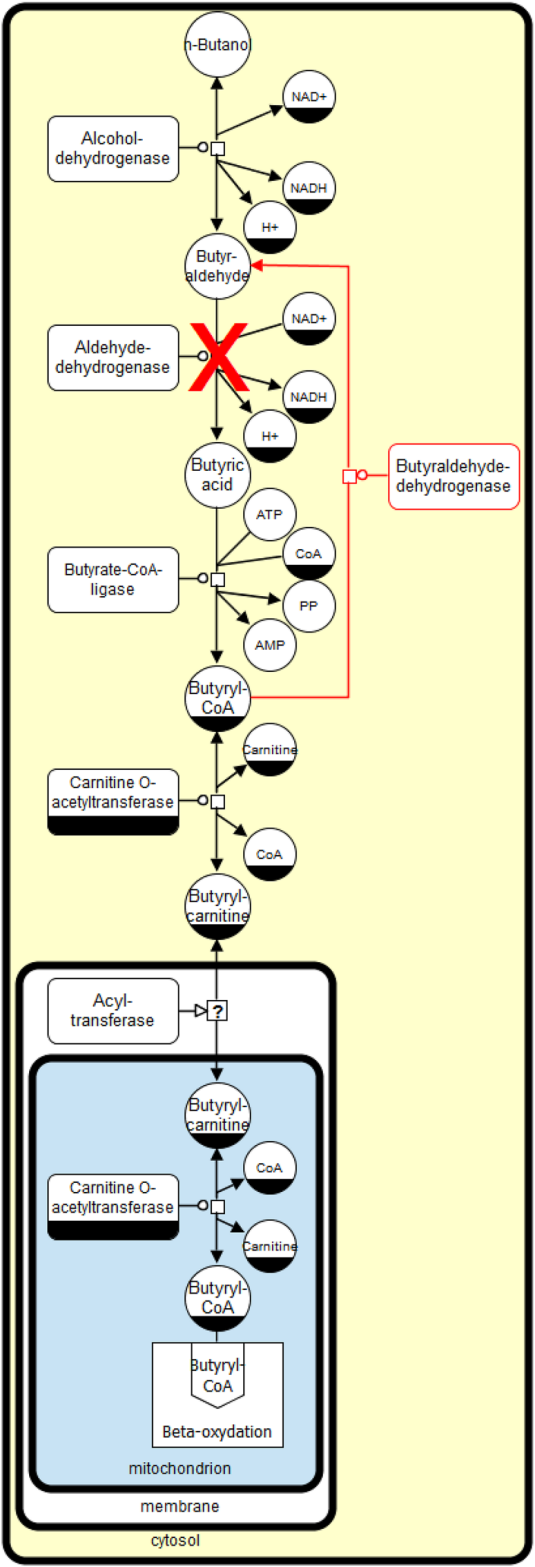
Schematic overview of the proposed 1-butanol degradation pathway in *A. adeninivorans*. The SBGN style metabolic network depicts reversible (*double headed arrow*) and irreversible (*single headed arrow*) reactions catalyzed by the corresponding enzymes (*rectangular square*). Metabolites or enzymes occurring multiple times in the metabolic network are decorated with a clone marker [e.g. NAD(P)^+^]. A strategy for 1-butanol production in *A. adeninivorans* is shown in *red* (produced using VANTED [2, 3])

The 1-butanol degradation pathway in the non-conventional, non-pathogenic yeast, *Arxula adeninivorans* (syn. *Blastobotrys adeninivorans*) was described by Kunze et al. [1]. Genome mining suggests that 1-butanol is oxidized by alcohol dehydrogenases and aldehyde dehydrogenases to butyric acid, ligated with CoA to form butyryl-CoA, and carnitine *O*-acetyltransferase to form butyryl-carnitine. The latter is then transported from the cytoplasm to the peroxisomes or mitochondria for β-oxidation. A special feature is the irreversible reaction from butyraldehyde to butyryl-CoA by aldehyde dehydrogenase and butyrate-CoA ligase (Fig. 1).

There has been speculation on which gene products are involved in this pathway. The first step is catalyzed by alcohol dehydrogenases and it is known that a further 18 gene products, are involved, making it particularly challenging to identify which of them participate in 1-butanol degradation [1].

Recently, increasing demand for 1-butanol as replacement for gasoline and as a fuel additive has renewed interest in the production of 1-butanol by fermentation [4]. *Clostridium acetobutylicum* [4, 5], metabolically engineered *Saccharomyces cerevisiae* [6], *Escherichia coli* [7] and *A. adeninivorans* have been used for its production [8].

*Arxula adeninivorans* required the addition of the *C. acetobutylicum ADHE2* gene coding for butyraldehyde dehydrogenase to overcome an irreversible step in the 1-butanol degradation pathway. Additionally, the knock-out of two *aldehyde dehydrogenase* genes was necessary to prevent degradation of the newly formed 1-butanol (Fig. 1, red). The equilibrium of butyraldehyde reduction also has to favour *A. adeninivorans* alcohol dehydrogenase (Aadhp) catalyzing the reduction of butyraldehyde to 1-butanol at a much faster rate than the oxidation of 1-butanol. It is thus necessary to improve the understanding of the Aadhp’s of *A. adeninivorans*, to which this paper makes a contribution. Aadhp1 favours butyraldehyde reduction with a three times smaller K_M_ compared to 1-butanol oxidation, a similar but low k_cat_ of 1300 min^−1^ (=21.7 s^−1^) and a higher, but still low, catalytic efficiency k_cat_/K_M_ of 810 min^−1^ mM^−1^ (=13.5 s^−1^ mM^−1^) [28].

In this work, the *Arxula ADH* gene, *AADH2*, was identified, isolated and constitutively expressed in *A. adeninivorans* by the Xplor^®^2 transformation and expression platform allowing the introduction of the AADH2 expression module either as yeast rDNA integrative expression cassette (YRC) or a yeast integrative expression cassette (YIC) into the yeast genome.

Recombinant Aadh2p was extracted from the cell by bead disruption, purified and biochemically characterized. The growth of the *AADH2* expressing yeast strain and a *∆aadh2* gene disruption mutant on different carbon sources were studied to elucidate the gene product’s role in *A. adeninivorans*.

## Results

### Identification of the *AADH2* gene of *A. adeninivorans* encoding Aadh2p involved in the 1-butanol degradation pathway

**Fig. 2:**
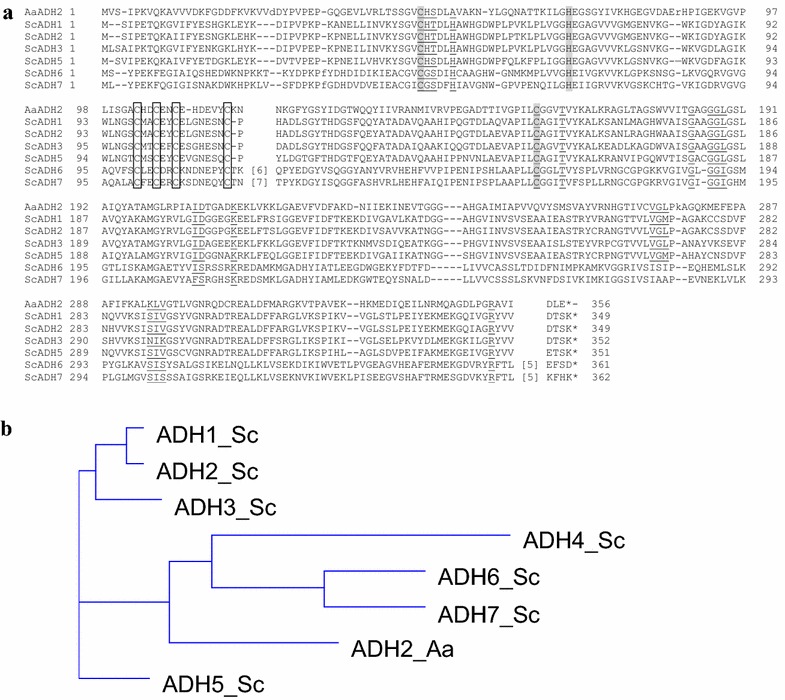
Alignment of Aadh2p sequence with the seven Adhp sequences from *S. cerevisiae* (ScAdhps). **a** Structural Zn^2+^ binding sites are shown framed [9–11], catalytic Zn^2+^ binding site in *grey* [9, 10] and NAD^+^ binding site is *underlined* [11–13]. **b** Phylogenetic tree constructed using the neighbour joining method of Aadh2p from *A. adeninivorans* (Aa) and Adhps from *S. cerevisiae* (Sc)

Nineteen genes were annotated as putative *AADH* genes in *A. adeninivorans*, of which nine were identified by sequence alignments with *S. cerevisiae ADH1* and *ADH2* genes. All had a score higher than 50. One of them had an open reading frame of 1068-bp encoding a 356 amino acid long protein (identity: 40 %, score: 204 and 208). This gene, designated Aadh2p had a calculated molecular mass of 38.9 kDa, which is in the same range as other ScAdhp monomers. It can be assigned to the cinnamyl alcohol dehydrogenases of the medium chain reductase/dehydrogenases family (MDR). Moreover, the NAD^+^ binding site and the Zn^2+^-binding domain for structural and catalytic Zn^2+^ was found by alignment with NCBI Blast (Fig. 2a) resulting in Aadh2p being identified as a Zn^2+^-binding NAD-dependent alcohol dehydrogenase.

A mitochondrial targeting sequence is absent, so it was presumed that the enzyme is localized in the cytoplasm. This hypothesis was confirmed by cell fractionation with sucrose gradient centrifugation (data not shown).

A phylogenetic tree between Aadh2p and ScAdhps from *S. cerevisiae* was developed using the neighbor joining method. It demonstrated that Aadh2p and ScAdh6p, 7p and 4p originate from the same ancestral node, whereas ScAdh1p, 2p, 3p and 5p are in two other branches (Fig. 2b).

### Generation of an Aadh2p producing yeast strain

**Fig. 3:**
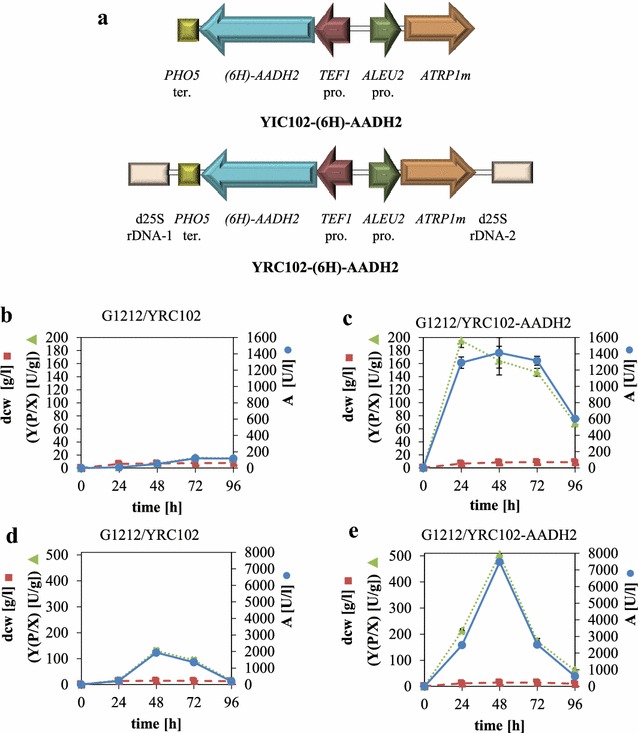
Physical maps of the YRC102-(6H)-AADH2 and YIC102-(6H)-AADH2 used for transformation of *A. adeninivorans* G1212 (**a**) and time-course traces of ADH activity by transgenic *A. adeninivorans* strains G1212/YRC102 and G1212/YRC102-AADH2 grown in various media (**b**–**e**). **a** Both cassettes contain the selection marker module with *ATRP1m* gene fused to the *ALEU2* promoter and the expression module with *TEF1* promoter—(*6H)*-*AADH2* gene—*PHO5* terminator. In addition YRCs (*Asc*I fragments) are flanked by 25S rDNA sequences for targeting, whereas YICs (*Sbf*I fragments) contain only the selection marker and expression modules. Transformants G1212/YRC102 (**b**, **d**) and G1212/YRC102-AADH2 (**c**, **e**) were cultured in shake-flasks for 96 h in YMM-glucose-NaNO_3_ at 30 °C (**b**, **c**) and YEPD at 30 °C (**d**, **e**). At the indicated times, 2 mL aliquots of the culture were used to determine biomass (dcw in g/L—*filled square*, *dashed line*) and to assay the intracellular Aadhp activity (U/L culture—*filled circle*, *solid line*) using butyraldehyde as substrate, and to calculate the Aadhp output Y (P/X) (U/g dcw—*filled triangle*, *dotted line*). For the determination of the dcw, 2 mL yeast culture was centrifuged and the pellet was washed with 1 mL water. The pellet was lyophilized and the tube with dried cells was weighted. Measurements were done in triplicate

The *AADH2* gene, without and with a polyhistidine-tag encoding sequence fused to the 5′-end of the ORF under the control of the strong constitutive *TEF1* promoter, was expressed in the auxotrophic mutant strain *A. adeninivorans* G1212 [Δ*atrp1*]. Cassettes with the AADH2 expression module (YRC102-6H-AADH2, YIC102-6H-AADH2, YRC102-AADH2, YIC102-AADH2—Fig. 3a) and control (YRC102) were prepared as described in “Methods” section. After integration of the cassettes into the genome, a number of selected clones (YICs and YRCs) were passaged to establish high plasmid stability. The transformants were then cultivated in YMM-glucose-NaNO_3_ at 30 °C for 48 h. The cells were harvested, disrupted and screened for ADH activity. ADH activity was detected in the *AADH2* and *6H*-*AADH2* expressing strains up to 12 times higher than in the control strain *A. adeninivorans* G1212/YRC102 using butyraldehyde as the substrate.

*AADH2* expression and control strains were grown on YMM-glucose-NaNO_3_ and YEPD at 30 °C for 96 h and the dcw, ADH activity (A) with butyraldehyde as the substrate and yield [Y(P/X)] were determined each day (Fig. 3b–e).

The maximal dcw was reached after 24 h in both media and then remained constant until the end of the experiment. The ADH activity found for *A. adeninivorans* G1212/YRC102-AADH2 was in all cases higher than for the control strain *A. adeninivorans* G1212/YRC102. In YMM-glucose-NaNO_3_ (Fig. 3c), maximum ADH activity of 1300 U/L was reached between 24 and 72 h with 200 U/g dcw for the *AADH2* overexpressing strains, while the activity for the control strain was consistently lower all the time of the experiment (Fig. 3b). In contrast, the ADH activity of *A. adeninivorans* G1212/YRC102 increased up to 2000 U/L and 120 U/g dcw after 48–72 h in YEPD medium (Fig. 3d). In this medium the ADH activity of the *AADH2* overexpressing yeast strain was highest after 48 h with 500 U/g dcw and 8000 U/L and decreased to 150 U/g dcw and 2000 U/L after a further 24 h incubation (Fig. 3e).

### Purification of 6 h-Aadh2p

*A. adeninivorans* G1212/YIC102-6H-AADH2 with the N-terminal His-tag was selected as the preferred yeast strain for the isolation and purification of recombinant 6h-Aadh2p, because the His-tag sequence at the C-terminus resulted in greatly reduced activity. The protein was purified by Ni–NTA (Novagen, Darmstadt, Germany) under native conditions with imidazole for elution as recommended by the manufacturer. Two elution steps were done each with 2.5 ml of elution buffer. After elution, these fractions were desalted with PD10 columns (GE Healthcare, Freiburg, Germany) to remove imidazole.

**Fig. 4:**
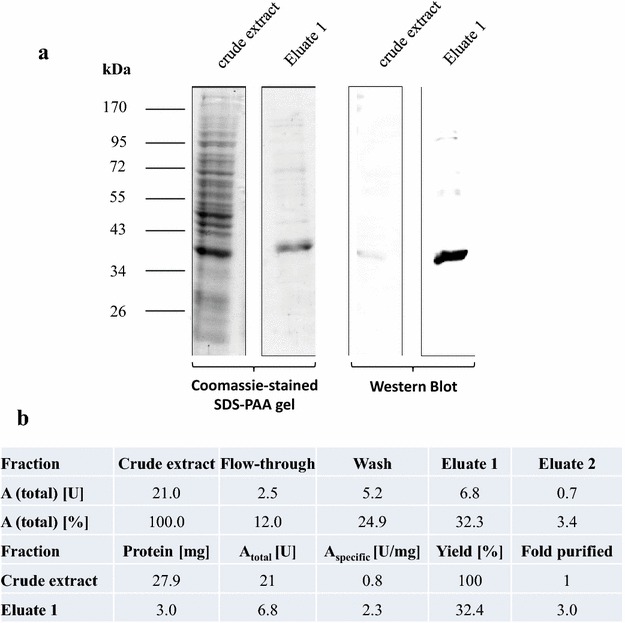
Purification of 6h-Aadh2p by Ni–NTA. **a** Coomassie-stained SDS-PAA gel and western Blot from crude extract (1) and eluate 1 (2) fractionated by electrophoresis on 12 % gel. The primary antibody was anti-poly Histidine from mice and the secondary antibody was anti-mouse IgG alkaline phosphatase. **b** Summary of 6h-Aadh2p purification. Intracellular soluble fraction of disrupted yeast cells from strain G1212/YIC102-6H-AADH2 was named ‘Crude extract’, proteins that did not bind to Ni–NTA were called ‘Flow-through’, proteins that were washed from the material was named ‘Wash’ and proteins that were eluted called ‘Eluate 1 and ‘Eluate 2′. Activity (A) was determined as described in the assay for determination of ADH activity

When 6h-Aadh2p was purified, 2.5 of 21 U were lost during the first binding step to Ni–NTA. A further 12.5 U were recovered in the washing fractions and it was assumed that this ADH activity comes from other Aadhp enzymes present in the *A. adeninivorans* crude extract. Approximately 8 U total (38 %) could be purified in two elution steps (Fig. 4a). In elution step one, 6.8 U were detected which is a yield of 32.4 %. There was a 3.03-fold concentration increasing the specific activity from 0.75 to 2.27 U/mg protein (Fig. 4b).

Analysis by western blotting showed a band around 39.3 kDa in the crude extract and elution step 1 (Fig. 4a), with the latter also visible in a Coomassie-stained SDS-PAA gel. The calculated molecular mass of 6h-Aadh2p is approximately 38.9 kDa.

### Properties of the recombinant 6h-Aadh2p

The optimum temperature and pH for ADH activity were investigated using purified Aadh2p. The following buffers were used to determine the optimal pH: 50 mM sodium acetate (pH 3–6), 50 mM sodium citrate (pH 3.5–6.5), 50 mM sodium phosphate (pH 5.5–8), 50 mM TRIS–HCl (pH 7–9). Highest activity detected for the reduction of butyraldehyde to 1-butanol was at pH 7–8 in sodium phosphate buffer whereas the oxidation of 1-butanol to butyraldehyde was maximal at pH 9 in TRIS–HCl. Thus, in all subsequent experiments, ADH reduction activity was determined in sodium phosphate pH 7.5 and oxidation activity determined in TRIS–HCl pH 9. The optimum temperature at pH 7.5 is 45 °C with 80 % of activity present between 38 to 48 °C.

Phosphate and acetate with monovalent cations are frequently used to buffer enzyme reactions e.g. sodium acetate, sodium phosphate and potassium phosphate. Here, sodium phosphate gave the highest enzyme activity. Ions such as Ca^2+^, Co^2+^, Cu^2+^, Fe^3+^, Mg^2+^, Mn^2+^, Ni^2+^, Zn^2+^ as chlorides were added at a concentration of 1 mM to the sodium phosphate/enzyme solution and reaction with butyraldehyde and NADH was followed to determine the effect of additional ions. Cu^2+^ inhibited the enzyme completely whereas Co^2+^ increased the relative activity up to 115 %. All other ions tested had no influence on the enzyme activity.

The approximate molecular mass of the native 6h-Aadh2p was detected by gel filtration on a Superdex™ 200 column. Recombinant maximum enzyme activity was confined to a peak with a M_r_ of 88 kDa. These results suggest that the most active form of 6h-Aadh2p is dimeric.

The kinetic constants of the purified protein were determined photometrically for NAD^+^, NADH, ethanol, 1-butanol, acetaldehyde and butyraldehyde as described in “Methods” section. The enzyme concentration for determination of apparent k_cat_ and k_cat_/K_M_ was estimated from a Coomassie-stained SDS-PAA gel of the protein. These constants are summarized in Table 1.

**Table 1:** Kinetic constants of 6h-Aadh2p synthesized in *A. adeninivorans* G1212/YIC102-6H-AADH2 for different substrates

Substrate	K_M_ [mM]	k_cat_ [1/s]	k_cat_/K_M_ [1/mM/s]
Ethanol^a^	14.2	1419	99.9
Acetaldehyde^b^	26.5	15.2	0.6
1-Butanol^a^	0.3	1312	4373
Butyraldehyde^c^	0.5	11872	23744
NAD^+d^	0.5	2.2	4
NADH^e^	0.07	14.7	224.5

^a^NAD^+^ was used in a concentration of 1 mM

^b^NADH was used in a concentration of 0.8 mM

^c^NADH was used in a concentration of 0.4 mM

^d^Ethanol was used in a concentration of 100 mM

^e^Acetaldehyde was used in a concentration of 20 mM

The K_M_ of the enzyme for ethanol and acetaldehyde is 46 or 53 times higher respectively than for 1-butanol and butyraldehyde. Turnover, k_cat_, for both alcohols is around 1500/s, whereas the catalytic efficiency, k_cat_/K_M_, is lower for acetaldehyde [0.6/(mM s)] compared to ethanol [99.9/(mM s)]. However, the efficiencies with four-carbon substrates (1-butanol, butyraldehyde) are much higher than with the two carbon substrates (ethanol, acetaldehyde), see Table 1.

The activity of 6h-Aadh2p was tested with different substrates for NAD^+^ dependent oxidation and NADH dependent reduction (Table 2). The highest oxidation activity found was for 1-butanol and was designated 100 %. The activity increased with the chain length from ethanol (70 %) to 1-butanol (100 %) and then decreased to 84 % for pentanol and hexanol and 33 % for 1-nonanol. Aromatic and primary alcohols are also oxidized by 6h-Aadh2p. There was almost no reaction with methanol and all secondary alcohols tested, except 2-butanol which had a relative activity of 23 %.

**Table 2:** Substrate specificity of 6h-Aadh2p synthesized in *A. adeninivorans* G1212/YIC102-6H-AADH2

Substrate	Relative enzyme activity [%]
NAD^+^-dependent oxidation	
Methanol	0.1
Ethanol	32
1-Butanol	100
1-Pentanol	84
1-Hexanol	84
Isopropanol	7
2-Butanol	23
1,2-Butanediol	2
2,5-Hexanediol	4
1-Phenylethanol	0
2-Phenylethanol	44
1-Nonanol^a^	33
2-Nonanol^a^	4
NADH-dependent reduction	
Acetaldehyde	36
Butyraldehyde	83
Pentanal	100
Hexanal	100
Acetone	0
2-Butanone	0
4-Hydroxy-3-butanone	0
5-Chlorpentanone	0
2,5-Hexanedione	0
Ethyl 4-chloro-acetate	0
Phenylacetaldehyde	83
Acetophenon	0
2-Nonanon^a^	0

Substrates were use at a concentration of 10 mM

^a^Substrates were used in a concentration of 1 mM

However the conversion of aldehydes to alcohols by NADH showed the opposite tendency. The highest activity was found with pentanol and hexanol as substrates and decreased to 83 % with butyraldehyde. Aromatic aldehydes are oxidized with a relative activity of 83 %, but there is no reaction with ketones.

If NADPH and NADP^+^ were used as cofactors, no activities were found for all substrates mentioned above.

### Expression analysis of *AADH2* on various carbon sources

**Fig. 5:**
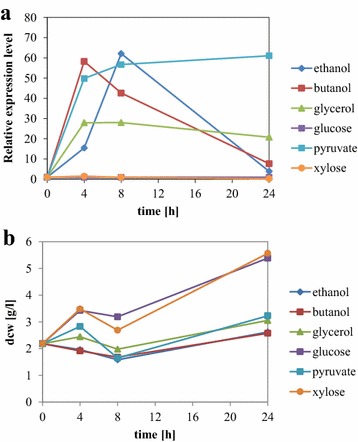
Influence of different carbon sources (2 % glucose, 2 % ethanol, 0.125 % 1-butanol, 2 % glycerol, 2 % pyruvate, 2 % xylose) on the gene expression levels of *AADH2* as well as the cell growth rate of *A. adeninivorans* LS3. **a** *A. adeninivorans* LS3 was cultivated on different carbon sources and at intervals, 2 mL culture was harvested, RNA isolated and expression analysis done by quantitative reverse transcriptase PCR analysis. Measurements were done in triplicate. **b** *A. adeninivorans* LS3 was cultivated on different carbon sources and at intervals, 2 mL culture was harvested and dcw was determined

Quantitative RT-PCR analysis was used to determine expression level of *AADH2* relative to the housekeeping genes *TFIID*, *ALG9* and *TFCI* [41, 42] of *A. adeninivorans* LS3 grown on different carbon sources (Fig. 5a). Dcw at different time points was determined (Fig. 5b). The influence of post-transcriptional changes was investigated, but only minor increases of enzyme activity were found when strains were grown on the different carbon sources and also for different periods (data not shown).

Cells cultured in ethanol, 1-butanol, glycerol or pyruvate showed much higher levels of expression 4 and 8 h after induction. After 4 h, RNA level of *AADH2* on 1-butanol increased to 58 times and decreased thereafter. Relative expression level on pyruvate is 61 times higher 4 to 24 h after induction, compared with glucose which is similar to the level of expression seen with ethanol. Glycerol induced (or derepressed) *AADH2* expression by up to 28 times. Xylose was the only substrate tested that had no effect on *AADH2* expression.

**Fig. 6:**
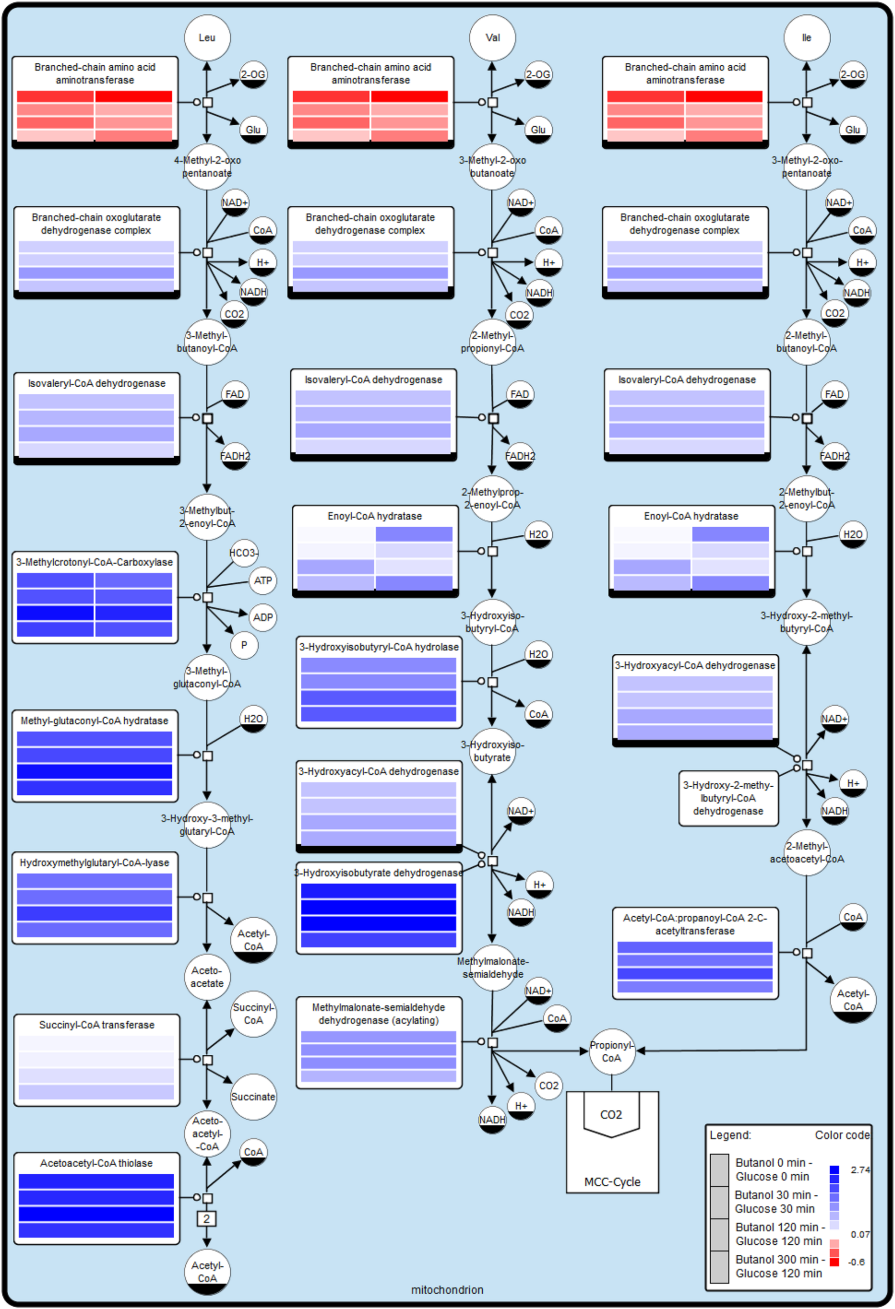
Key compounds of the leucine, valine and isoleucine metabolism—microarray studies. The SBGN style metabolic network depicts reversible (*double headed arrow*) and irreversible (*single headed arrow*) reactions catalyzed by the corresponding enzymes (*rectangular square*). Enzymes are enriched with color-coded fold change values of time resolved expression data of the respective genes. The colors represent upregulation (*blue*) and downregulation (*red*) of genes in cells shifted to medium containing 1-butanol as the carbon source compared to cells grown with glucose. Metabolites or enzymes occurring multiple times in the metabolic network are decorated with a clone marker (e.g. NAD^+^) (produced using VANTED [2, 3])

Microarrays based on the complete genome data of *A. adeninivorans* were used to analyze changes in gene expression before and after a shift of *A. adeninivorans* LS3 from YMM-glucose-NaNO_3_ to YMM-glucose-NaNO_3_ containing 0.125 % 1-butanol. Cells were harvested after 15, 30 min, 2 and 5 h of shaking at 30 °C and 180 rpm and total RNA was isolated. As well as modification in the glyoxylate cycle, methyl citrate cycle and ß-oxidation (Additional file 1: Figure S1, Additional file 2: Figure S2, Additional file 3: Figure S3), a significant upregulation of the genes involved in the catabolism of the branched-chain amino acids valine, leucine and isoleucine was observed directly after the shift. In contrast, the key component of the branched-chain amino acids metabolism pathway, the branched-chain amino acid aminotransferase encoding gene, was down regulated (Fig. 6). Microarray data also suggests regulation of other putative alcohol and aldehyde dehydrogenase genes occurs after the shift to a 1-butanol containing medium. Experimental verification of these results will provide further insight into the regulation of 1-butanol degradation by *A. adeninivorans* and indicates the possibility of using transcriptomic approaches to identify candidate genes for new biotechnological applications.

### Role of Aadh2p in *A. adeninivorans* on metabolizing glucose, ethanol and 1-butanol

It was found that *AADH2* gene is upregulated when wild-type *A. adeninivorans* strain LS3 is growing on 1-butanol, ethanol, pyruvate and glycerol but the role of Aadh2p in related metabolic pathways is still unknown.

**Fig. 7:**
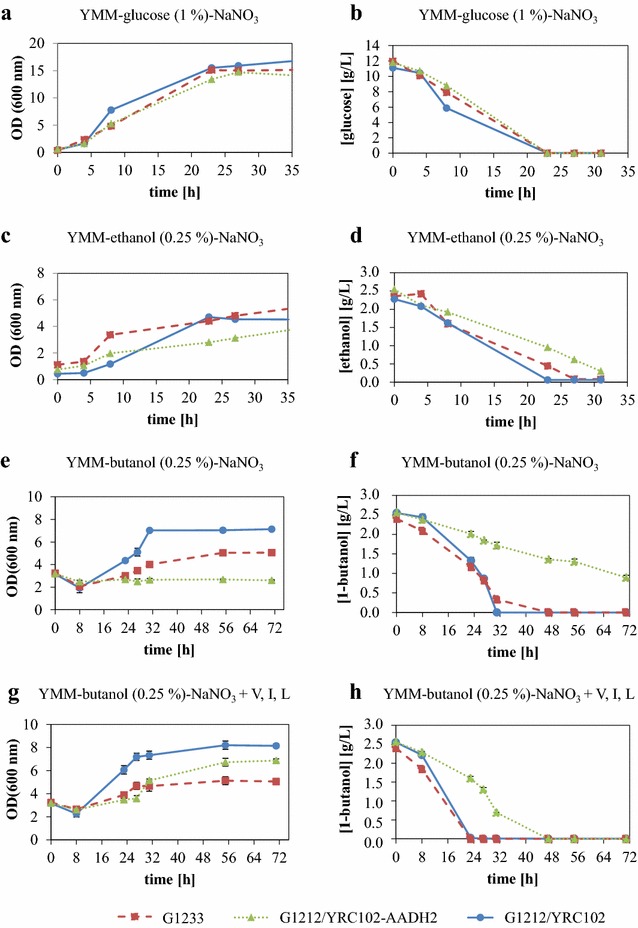
Analysis of G1212/YRC102, Δ*aadh2* mutant G1233 and G1212/YRC102-AADH2 cultured on YMM-glucose (2 %)-NaNO_3_ (**a**–**b**), YMM-ethanol (0.25 %)-NaNO_3_ (**c**–**d**) or YMM-1-butanol (0.25 %)-NaNO_3_, the latter with (**e**–**f**) and without (**g**–**h**) valine, leucine and isoleucine in the medium. **a**, **c**, **e**, **g** Time-course plots of optical density at 600 nm; **b** glucose or **d** ethanol or **f**, **h** 1-butanol concentration in the culture medium of transgenic *A. adeninivorans* strains G1212/YRC102 (*filled circle*, *solid line*), G1233 (*filled rectangle*, *dashed line*) and G1212/YRC102-AADH2 (*filled triangle*, *dotted line*). Measurements were done in triplicate

Experiments concerning growth on different carbon sources were undertaken with *A. adeninivorans* strains G1212/YRC102, G1233 (Δ*aadh2*) and G1212/YRC102-AADH2. They were grown on YMM-glucose-NaNO_3_ for 24 h and shifted to YMM-glucose (1 %)-NaNO_3_, YMM-ethanol (0.25 %)-NaNO_3_ or YMM-1-butanol (0.25 %)-NaNO_3_ (Fig. 7).

The mutant strain G1233 had only approximately 50–60 % ADH activity of the control strain G1212/YRC102 when cultivated on ethanol or 1-butanol (data not shown).

All strains show a similar glucose degradation profile and cell growth. The OD_600 nm_ increases from 0.5 to 15 over 23 h and then stays constant, because glucose has been completely consumed (Fig. 7a, b). On ethanol, the OD_600 nm_ increases from 0.5 to 4.4 over 23 h for G1212/YRC102 or 27 h for G1233 in which ethanol is completely depleted. Overexpression of the *AADH2* gene led to lower rate of consumption of ethanol over more than 32 h and a decrease in the growth rate compared to control strain and the knock-out mutant strain G1233 (Fig. 7c, d).

The OD_600 nm_ of *A. adeninivorans* G1212/YRC102 incubated with 1-butanol did not start to increase until 8 h, but then the OD_600 nm_ increased continuously to 7 until the substrate was consumed completely at 32 h (Fig. 7e, f).

In contrast *A. adeninivorans* G1233 (Δ*aadh2*) showed slower growth on YMM-1-butanol (0.25 %)-NaNO_3_ compared with the control strain with a maximum OD_600 nm_ of 5 and total consumption of 1-butanol achieved after 56 h cultivation.

G1212/YRC102-AADH2 expressing the *AADH2* gene controlled by the strong constitutive *TEF1* promoter did not start to grow with 1-butanol as a sole carbon source until 72 h. GC–MS analysis showed that 1-butanol concentration then slowly decreased in the medium (Fig. 7e, f).

The addition of valine, leucine and isoleucine to YMM-1-butanol (0.25 %)-NaNO_3_ enabled the growth of the AADH2-overexpressing transformants to reach a maximum OD_600 nm_ of 7 at 56 h. Growth behavior of G1212/YRC102 and G1233 was unchanged by addition of the branched-chain amino acids except that the maximum OD_600 nm_ for G1212/YRC102 increased from 7 to 8 (Fig. 7g).

Valine, leucine and isoleucine accelerated 1-butanol degradation, which was completely consumed after 24 h by G1212/YRC102 and G1233 and at 48 h for G1212/YRC102-AADH2 (Fig. 7h). Thus, it is conceivable that genes were induced or repressed by 1-butanol and influenced the degradation of these branched amino acids.

ADH activity for any given optical density for the AADH2 transformant, G1212/YRC102-AADH2, was around 6 times higher when branched-chain amino acids were added to YMM-1-butanol (0.25 %)-NaNO_3_ and was comparable with activity found with cultivation on glucose.

## Discussion

Seven *ADH* genes have been identified in *S. cerevisiae* (*ScADH*) and five encode enzymes involved in ethanol metabolism. ScAdh1p, ScAdh3p, ScAdh4p and ScAdh5p reduce acetaldehyde to ethanol, whereas ScAdh2p catalyzes the reverse reaction [14–18]. All ScAdhps, and Adhps from other yeasts are very similar among each other, so that regulation and physiological function are not predictable. However, knowledge of the function of the different Aadhps would provide useful insights into the 1-butanol degradation pathway and contribute information necessary to understand 1-butanol synthesis in genetically manipulated *A. adeninivorans* strains [1].

In this study the *AADH2* gene of *A. adeninivorans* was overexpressed in a host cell and the molecular mass, optimum reaction conditions, kinetic characteristics, and substrates for the enzyme were characterized.

Aadh2p shares an identity of 40 % with ScAdh1p and ScAdh2p [19], which are required for the reduction of acetaldehyde to ethanol during glucose fermentation (ScAdh1p) or for ethanol oxidation to acetaldehyde during ethanol catabolism (ScAdh2p).

Although Aadh2p clusters with ScAdh6p, 7p and 4p according to Fig. 2b, identities are only 22.9, 25.5 and 13.7 % respectively. Aadh2p and ScAdh6p/7p belong to the medium chain alcohol dehydrogenases and are members of the cinnamyl family of alcohol dehydrogenases, which possess Zn^2+^ binding domains. However they differ in their cofactor preference [19] with ScAdh6p and 7p using NADP(H) as cofactors [19], whereas Aadh2p and ScAdh4p use NAD(H) [17]. Another difference is that Aadh2p is localized in the cytoplasm whereas ScAdh4p, is located in mitochondria [17].

The Aadhp from overexpression of *AADH2* in *A. adeninivorans* had activities of up to 8000 U/L culture (500 U/g dcw) in YEPD after 48 h cultivation, which is 4 times higher than for the control strain. However growth in YMM-glucose-NaNO_3_ was restricted and resulted in low levels of recombinant Aadh2p.

Purified 6h-Aadh2p catalyzed the reduction of aldehydes at pH 7.5 and oxidation of alcohols at pH 9.0 (both at 45 °C). Verduyn et al. [20] also found that ADH oxidation activity was best at approximately pH 9.0 in *Candida utilis*, *Ogataea polymorpha* and *S. cerevisiae.*

Primary alcohols are the preferred substrates for 6h-Aadh2p with 1-butanol giving the highest activity. The enzyme was less reactive with longer and shorter chain alcohols which is similar to the activities of ScAdh6p and 7p, which had their highest activities with 1-pentanol and 1-hexanol [21, 22]. The 6h-Aadh2p Michaelis–Menten constants for ethanol and acetaldehyde are higher than those for 1-butanol and butyraldehyde. 6h-Aadh2p’s K_M_ for ethanol is 14.2 mM which is seven times higher than that for 6h-Aadh1p, whereas the reverse was seen for 1-butanol with a K_M_ for 6h-Aadh2p of 0.3 mM which is more than five times smaller than that of 6h-Aadh1p [28]. The catalytic efficiency of 23,744/(mM s) for butyraldehyde is 5 times greater than it is with 1-butanol and is nearly 6000 times higher than that of 6h-Aadh1p [4/(mM s)]. Furthermore the k_cat_/K_M_ for 6h-Aadh2p with 1-butanol exceeds that of 6h-Aadh1p by 324-fold [28]. The much higher catalytic efficiency found for butyraldehyde compared to 1-butanol is similar to those found for ScAdh6p and 7p, which have 5356 and 262 times higher k_cat_/K_M_ for hexanol and pentanol compared to 1-hexanol and 1-pentanol respectively [21, 22].

The K_M_ of 14.2 mM for ethanol is higher than that of 6h-Aadh1p (2 mM) and the K_M_ for acetaldehyde is 26.5 mM, which is double in comparison with 6h-Aadh1p. The turnover number of ethanol oxidation is 87 times higher than that for 6h-Aadh1p (16.3/s). The catalytic efficiency of 99.9/(mM s) is twice as high as for 6h-Aadh1p. However, k_cat_ and k_cat_/K_M_ for acetaldehyde of 15.2/s and 0.6/(mM s) are much lower than that of 6h-Aadh1p [98.3/s, 6.7/(mM s)] [28]. This indicates that 1-butanol oxidation and butyraldehyde reduction are much more effective if 6h-Aadh2p is used as biocatalyst, compared to 6h-Aadh1p, however if ethanol and acetaldehyde are the substrates these results are reversed.

Expression analysis of *AADH2* revealed it was induced or derepressed on non-fermentable carbon sources such as 1-butanol, ethanol, glycerol and pyruvate when compared to growth on glucose, by up to 63-fold, whereas there were no differences in expression level for cultivation under the same conditions on the fermentable carbon-source xylose.

Aadh2p could also participate in aerobic alcoholic fermentation (Crabtree effect), because pyruvate decarboxylase can dissimilate high pyruvate concentrations and trigger alcoholic fermentation [23, 24]. Furthermore, Aadh2p is positively regulated by glycerol as was found for the ADH I of *A. nidulans* [25].

Growth studies of control strain G1212/YRC102, knock-out mutant G1233 and overexpression strain G1212/YRC102-AADH2 on glucose, ethanol and 1-butanol provided further information on the role of Aadh2p. There were no differences in growth and glucose consumption rates among the three different yeast strains which agrees with the level of expression detected in analysis of the *AADH2* gene.

While Aadh2p is involved in ethanol degradation as found by quantitative PCR, knock-out or overexpression of the gene had only minor effects on cell growth during cultivation on ethanol (Fig. 7c). Thus it is likely that there are other ADH isozymes, which can substitute for Aadh2p. This has been observed in *Scheffersomyces stipitis* and *O. polymorpha* which can grow on ethanol despite ADH disruption [26, 27].

When 1-butanol is used as carbon source, genes for branched-chain amino acids catabolism are upregulated and genes for their metabolism are down-regulated. If the Aadh2p level is in right balance as it is in G1212/YRC102, 1-butanol can be degraded preferentially and thus the influence on valine, leucine and isoleucine degradation is limited.

If the *AADH2* gene is knocked-out, 1-butanol is not as effectively degraded as in G1212/YRC102, meaning that 1-butanol is consumed more slowly, which leads to stronger induction of branched-chain amino acid degradation and inhibition of their production.

The overexpression of the *AADH2* gene leads to a shift of the equilibrium toward production of 1-butanol and away from its consumption. Thus valine, leucine and isoleucine catabolism is switched-on over a long period of time and the amino acids are assumed to be absent in the cell. The cells do not divide but remain viable and if valine, leucine and isoleucine are added to the medium, the cells resume growth (data not shown).

Aadh1p and Aadh2p have similar characteristics. Both favour short and medium chain length alcohols and aldehydes as substrates, are localized in the cytoplasm and use NAD^+^/NADH as cofactors. Aadh2p shares 40 % identity with *S. cerevisiae* ADH1, whereas Aadh1p has 64 % identity [28]. However there are some differences between these enzymes. Aadh2p favours 1-butanol as a substrate, whereas Aadh1p oxidizes ethanol with highest activity. Expression levels of the *AADH2* gene on ethanol, 1-butanol, pyruvate and xylose [28] are much higher with an increase of up to 60 times, whereas for the *AADH1* gene the increase in expression on the same substrates is a maximum of 2 times.

Furthermore overexpression of *AADH1* led to a slight increase in 1-butanol degradation, whereas higher levels of Aadh2p in the cells inhibited their growth and 1-butanol consumption. To conclude Aadh1p is predominantly important for ethanol degradation and synthesis and has only minor effects on 1-butanol metabolism [28], whereas Aadh2p plays major role in 1-butanol metabolism.

## Conclusions

Here, we report the overexpression of an *AADH* gene from *A. adeninivorans* (*AADH2*). The resulting Aadh2p operates in conjunction with other enzymes involved in the 1-butanol metabolism. The enzyme has high affinities and catalytic activities for butyraldehyde and 1-butanol compared to those for acetaldehyde and ethanol. It was also found that Aadh2p favours butyraldehyde reduction over 1-butanol oxidation. Gene expression is upregulated by non-fermentable carbon sources such as ethanol, pyruvate, glycerol and 1-butanol and 1-butanol seems to induce genes necessary for the degradation of branched-chain keto acids limiting the availability of these amino acids. This combined with the primary Aadh2p reaction of converting butyraldehyde to 1-butanol explains why the overexpression of *AADH2* gene prevents cell growth and why this effect can be overcome by addition of leucine, valine and isoleucine. Thus the constitutive expression of the *AADH2* gene may be useful in the production of 1-butanol by *A. adeninivorans,* although it is likely that other *ADHs* will have to be knocked-out to prevent 1-butanol oxidation.

## Methods

### Strains and culture conditions

*E. coli* XL1 blue [*recA1, endA1, gyrA96, thi*-*1, hsdR17, supE44, relA1, lac* [F´*proABlacl* q Z DM15 Tn10 (Tetr)] obtained from invitrogen (USA), served as the host strain for bacterial transformation and plasmid isolation. The strain was grown on LB medium (Sigma, USA) supplemented with 75 mg/L ampicillin (Applichem, Germany) or 75 mg/L kanamycin (Roth, Germany) for selection.

In this study, the auxotrophic mutant *A. adeninivorans* G1212 (*aleu2 ALEU2::atrp1—*[29]) and the wild-type *A. adeninivorans* strain LS3 were used. LS3 was originally isolated from wood hydrolysates in Siberia and deposited as *A. adeninivorans* SBUG 724 in the strain collection of the Department of Biology of the University of Greifswald [30]. All strains were grown at 30 °C under non-selective conditions in a complex medium (YEPD) or under selective conditions in yeast minimal medium supplemented with 2 % (*w/v*) glucose as a carbon source and 43 mM NaNO_3_ as a nitrogen source unless stated otherwise (YMM-glucose-NaNO_3_) [31, 32].

Agar plates were prepared by adding 1.6 % (*w/v*) agar to the liquid medium.

### Transformation procedures and isolation of nucleic acids

Transformation of *E. coli* was performed according to Hanahan [33] and *A. adeninivorans* cells were transformed according to Böer et al. [34]. Isolation of plasmid and chromosomal DNA and DNA restrictions were carried out as previously described [35].

### Construction of AADH2 expression plasmids and generation of transgenic *A. adeninivorans* strains

For *AADH2* overexpression, the *AADH2* ORF (LN828975) without a His-tag and with a His-tag encoding region at the 5′ end (*6H*-*AADH2*) were amplified from a chromosomal DNA template in a PCR reaction using primers that incorporated flanking *Eco*RI and *Bam*HI cleavage sites (primer AADH2-1-6H–5′-GCC**GAATTC**ATGCACCATCATCACCACCACGTTCCATCCCCCGATATT-3′; primer AADH2-1 – 5´-GCC**GAATTC**ATGGTTCCATCCCCCGATATT-3′, nucleotide positions 1-21, *Eco*RI restriction site is in bold type and underlined; primer AADH2-2–5′-GCG**GGATCC**TTATTCAAGATCAATGACTGCAC-3′, nucleotide positions 1046-1068, *Bam*HI restriction site is in bold type and underlined). The resulting *Eco*RI-*Bam*HI flanked (*6H)*-*AADH2* ORF was inserted into the plasmid pBS-TEF1-PHO5-SS between the *A. adeninivorans* derived *TEF1* promoter and the *S. cerevisiae*-derived *PHO5* terminator [36]. The *TEF1* promoter—(*6H)*-*AADH2* ORF – *PHO5* terminator flanked by *Spe*I-*Sac*II restriction sites expression modules was inserted into the plasmid Xplor2.2 to generate Xplor2.2-TEF1-(6H)-AADH2-PHO5. The resulting plasmids contain fragments of 25S rDNA, which are interrupted by the selection marker module (*ALEU2* promoter—*ATRP1* *m* gene—*ATRP1* terminator—[29]), the expression module and an *E. coli* resistance marker. To prepare the cassettes for yeast transformation, Xplor2.2-TEF1-(6H)-AADH2-PHO5 and the control plasmid Xplor2.2 lacking AADH2 expression module were digested with *Asc*I (YRC) and/or *Sbf*I (YIC) to remove the *E. coli* sequences including the resistance marker. The resulting restriction products YRC102-6H-AADH2, YIC102-6H-AADH2, YRC102-AADH2, YIC102-AADH2 and YRC102 (control) were used to transform *A. adeninivorans* G1212.

Yeast transformants were selected by tryptophan auxotrophy in YMM-glucose-NaNO_3_. The cells were then stabilized by passaging on selective (YMM-glucose-NaNO_3_) and non-selective (YEPD) agar plates to attain a high level of protein production [37].

### Construction of *A. adeninivorans* Δ*aadh2* gene disruption mutant

Fragments of approximately 1000-bp, located at the 5′ and 3′ ends of the open reading frame (ORF), were amplified by PCR with chromosomal DNA from *A. adeninivorans* LS3 as template. The primer combinations, AADH2-3 (5′-CTCAGCTCTATCTCTGCCT-3′, position numbers −1291 to −1273) and AADH2-4 (5′-GAG**TCCGGA**CTATACTTGTTTGGAGCGC-3′, position numbers −344 to −326, *Kpn*2I restriction site in underlined bold type) were used for amplification of the 5´ region and AADH2-5 (5′-GCG**TCCGGA**ATCGTCACTGTCATATCACA-3′, position numbers 1158–1177, *Kpn*2I restriction site in underlined bold type) and AADH2-6 (5′-CAATTCAATGTTGCGATT-3′, position numbers 2227–2244) were used to amplify the 3´ region. After ligation of the 5´ region with the 3´ region (separated by *Kpn*2I restriction site), the 2058-bp fragment was inserted into the *E. coli* vector pCR4 (Invitrogen, USA). Subsequently the selection marker *ATRP1* *m,* flanked by *Kpn*2I, was inserted between the 5´and 3´regions at both ends of the *AADH2* gene by *Kpn*2I restriction and ligation. Finally, the complete construct covering 1000-bp in front of the *AADH2* gene, the ATRP1m selection marker module and 1000-bp behind the *AADH2* gene was amplified using the primers AADH2-3 and AADH2-6. The resulting 3196-bp PCR-product was used to transform *A. adeninivorans* G1212 [35].

### Assay for determination of ADH activity

The assay for the determination of the ADH activity was performed in 50 mM sodium phosphate buffer pH 7.5 containing butyraldehyde (10 mM, Sigma-Aldrich) and NADH (0.4 mM, Roth) for reduction or 50 mM TRIS–HCl pH 9.0 with 1-butanol (10 mM, Sigma-Aldrich) and NAD^+^ (1 mM, Roth) for oxidation. The reaction was started by the addition of the enzyme solution and monitored for a minimum 2.5 min at 340 nm, 30 °C. The change in absorbance was then converted to oxidized NADH or reduced NAD^+^ concentration using the Beer–Lambert equation (ε = 6200 M^−1^ cm^−1^ for NADH). One Unit (1 U) of enzyme activity was defined as the amount of enzyme required to oxidize 1 µmol NADH to NAD^+^ or reduce 1 μmol NAD^+^ to NADH per min at 30 °C, pH 7.5 or 9.0.

### Determination of molecular mass and K_M_ of Aadh2p

The determination of the Aadh2p molecular mass was done by gel filtration using Superdex™ 200 (Amersham Biosciences, UK). The flow rate was 1 mL/min and fractions of about 1 mL were collected for 152 min (buffer: 50 mM sodium phosphate pH 6.5 + 0.15 M NaCl). A calibration curve was constructed using bovine serum albumin, ovalbumin, catalase and alcohol dehydrogenase from *S. cerevisiae* as standards.

The K_M_ value for the substrates: ethanol, 1-butanol, NAD^+^, acetaldehyde, butyraldehyde and NADH, were determined as described in ‘Assay for determination of ADH-activity’. All determinations were done in triplicate and Michaelis–Menten and Hanes plots were constructed.

The Aadh2p concentration was determined using a Coomassie stained SDS-PAA gel for the calculation of k_cat_ [38].

### Protein analysis

SDS-PAGE and western analysis were performed as described by Kunze et al. [37]. Western blots were treated with an anti-His-tag specific primary antibody produced in mice (Sigma-Aldrich, Germany) and a rabbit anti-mouse IgG alkaline phosphatase conjugate (Sigma-Aldrich, USA), and subsequently stained by incubation with NBT/BCIP substrate (Roche Diagnostics, Switzerland).

The dye-binding method of Bradford [39] was used for protein quantification (BIO-RAD, USA), using bovine serum albumin as the standard.

### Quantitative reverse transcriptase PCR analysis

*A. adeninivorans* LS3 cells were grown in YMM-glucose-NaNO_3_ for 24 h at 30 °C and 180 rpm. Cells were harvested (3220×*g* at 4 °C), washed with YMM-NaNO_3_ and washed cells were resuspended in YMM-NaNO_3_ with different carbon sources (2 % glucose, 2 % ethanol, 0.125 % 1-butanol, 2 % glycerol, 2 % pyruvate, 2 % xylose) at an OD_600 nm_ of 3. After 1, 4, 8 and 24 h, 2 mL of culture was harvested (2300×*g*, 4 °C), RNA was isolated using RNeasy Mini Kit (Qiagen) as described by the manufacturer including mechanical disruption of the cells using silica beads and a Mixer Mill MM400 (RETSCH) operating for 3 min, vibrational frequency of 30/s. RNA was analysed using denaturating agarose gel electrophoresis. A first strand cDNA was synthesized using RevertAid H Minus First Strand cDNA Synthesis Kit (Fermentas) with Oligo(dT) 15 V-RTA primer. The first PCR synthesis was made with cDNA-template (10 cycles) using gene-specific primer 1 or 3 and RTA1 primer (for TFCI primer 1 or 2 was used). The PCR product was amplified in the presence of SYBR Green fluorophore (Power SYBR^®^ Green PCR Master Mix, Applied Biosystems, Foster City, CA, USA) using ABI 7900HT Fast Sequence Detection System (Applied Biosystems) with gene-specific primer 2 or 4 and RTA primer (for TFCI primer 2 or 3 was used). Primers are listed in Table 3. *TFIID*, *ALG9* and *TFCI* were used as reference (housekeeping) genes [40, 41]. Primer efficiencies were >1.98 and calculations were done using ΔΔc_t_-method [42].

**Table 3:** Sequences of primers used for analysis of *AADH2* expression levels

Primer	Sequence (5′→3′)
Oligo(dT)15 V-RTA	TGACAGGATACCATACAGACACTATTTTTTTTTTTTTTTV-wobbles
RTA-1	TGACAGGATACCATACAGACAC
RTA	TGACAGGATACCATACAGACACTA
ADH2-1	TATGCAGGCCGGTGATCT
ADH2-2	GGACGTGCAGTCATTGATC
ADH2-3	ATTCTCAACCGTATGCAGG
ADH2-4	GATCTTCCTGGACGTGCA
ALG9-1	CATGGGCCAAGGTATACTG
ALG9-2	GAAAAAGTGCCCAAACGA
ALG9-3	TGGTATCGGTCGCATTCT
ALG9-4	TCAATTGCAGTGGACTGACTA
TFllD-1	ACGAGCGGTACTCACAATG
TFllD-2	ATGGACTCAATGTCAAACGAC
TFllD-3	AGTTTGTGTCTGATATTGCCTC
TFllD-4	TGGCCGATTATGGACTCA
TFC1-1	TGAAGAAGAGCACCAAGCA
TFC1-2	ACAACAAGATGAAAACGC
TFC1-3	ATGATGACGATGATGACGAAT

### Microarray design and hybridization for gene expression analysis

Based on 6025 annotated chromosomal sequences and 36 putative mitochondrial genes oligos were designed using Agilent Technologies eArray software (https://earray.chem.agilent.com; design number 035454). Depending on the sequence length of the genes up to 10 60-mers per gene were created resulting in a total of 56,312 *A. adeninivorans* specific oligos. The microarray was produced by Agilent Technologies in 8 × 60 k format.

Overnight cultures of *A. adeninivorans* LS3 in YMM-glucose-NaNO_3_ were shifted to YMM-glucose-NaNO_3_ with 0.125 % 1-butanol and YMM-glucose-NaNO_3_. After 15, 30 min, 2 and 5 h of shaking at 30 °C and 180 rpm, cells were harvested and total RNA was isolated. Probe labeling and microarray hybridization (duplicates) were executed according to the manufacturer’s instructions (Agilent Technologies “One-Color Microarray-Based Gene Expression Analysis”; v6.5).

Analysis of microarray data was performed with the R package limma [43]. Raw expression values were background corrected using “normexp” and normalized between arrays using “quantile”. Differentially expressed genes were detected by fitting a linear model to log2-transformed data by an empirical Bayes method [44]. The Bonferroni method was used to correct for multiple testing.

### Analysis of supernatant from strains growing on YMM-glucose (1 %)-NaNO_3_, YMM-1-butanol (0.25 %)-NaNO_3_ and YMM-ethanol (0.25 %)-NaNO_3_

Glucose concentration was measured with the 3,5-dinitrosalicylic acid (DNSS) assay. 100 µL of supernatant, diluted if necessary, was mixed with 100 µL DNSS reagent (44 mM DNSS, 0.4 M sodium hydroxide, 0.4 M potassium hydroxide and 1 M potassium sodium tartrate in distilled water), heated for 15 min at 99 °C and centrifuged (1 min, 5000×*g*). The supernatant (100 µL) was transferred to 96-well microtiter plate and absorption was measured at 530 nm (measurement wavelength) and 600 nm (reference wavelength). A calibration curve of different glucose concentrations versus absorption at 530 nm minus absorption at 600 nm was plotted and used for calculating glucose concentrations in the samples.

1-Butanol concentration of *A. adeninivorans* culture was directly measured by GC–MS (Clarus SQ 8 GC Mass Spectrometers; 1 µL sample, column: Elite-624, length: 30 m, I.D: 0.25 mm, film thickness: 1.40 µm, Perkin Elmer, Germany) after centrifugation for 5 min, 16,000×*g*. The temperature was held at 60 °C for 10 min and was then ramped up to 230 °C at 15 °C/min and held at this temperature for 3 min.

The ethanol content of the culture supernatant was quantified by Headspace GC–MS (Perkin Elmer Turbo-Matrix 40 Headspace Sampler with Clarus GC680 and SQ8S mass spectrometer). The sample was centrifuged for 1 min at 16,000×*g* in a micro-centrifuge and 10 µL supernatant transferred to a 22.3 mL Headspace and thermostatted at 130 °C for 30 min. After pressurizing for 1 min and a 0.05 min withdrawal period, the sample was separated isothermally at 60 °C on a Elite 624 column (length: 30 m, I.D: 0.25 mm, film thickness: 1.40 µm, Perkin Elmer, Germany). Sample detection was carried out in TIC mode in triplicate with external quantification standards assayed four times.

## Additional files

10.1186/s12934-016-0573-9 Key compounds of the glyoxylate cycle—microarray studies. The SBGN style metabolic network depicts reversible (double headed arrow) and irreversible (single headed arrow) reactions catalyzed by the corresponding enzymes (rectangular square). Enzymes are enriched with color-coded fold change values of time resolved expression data of the respective genes. The colors represent upregulation (blue) and downregulation (red) of genes in cells shifted to medium containing 1-butanol as the carbon source compared to cells grown with glucose. Metabolites or enzymes occurring multiple times in the metabolic network are decorated with a clone marker (e.g. CoA) (produced using VANTED [2, 3]).

10.1186/s12934-016-0573-9 Key compounds of the methyl citrate cycle—microarray studies. The SBGN style metabolic network depicts reversible (double headed arrow) and irreversible (single headed arrow) reactions catalyzed by the corresponding enzymes (rectangular square). Enzymes are enriched with colour-coded fold change values of time resolved expression data of the respective genes. The colours represent upregulation (blue) and downregulation (red) of genes in cells shifted to medium containing 1-butanol as the carbon source compared to cells grown with glucose (produced using VANTED [2, 3]).

10.1186/s12934-016-0573-9 Key compounds of the ß-oxidation - microarray studies. The SBGN style metabolic network depicts reversible (double headed arrow) and irreversible (single headed arrow) reactions catalyzed by the corresponding enzymes (rectangular square). Enzymes are enriched with color-coded fold change values of time resolved expression data of the respective genes. The colors represent upregulation (blue) and downregulation (red) of genes in cells shifted to medium containing 1-butanol as the carbon source compared to cells grown with glucose. Metabolites or enzymes occurring multiple times in the metabolic network are decorated with a clone marker (e.g. CoA) (produced using VANTED [2, 3]).

## Abbreviations

ADH, adhp: alcohol dehydrogenases
*AADH1* gene: *alcohol dehydrogenase 1* gene from *Arxula adeninivorans*
Aadh1p: alcohol dehydrogenase 1 protein from *Arxula adeninivorans*
*AADH2* gene: *alcohol dehydrogenase* 2 gene from *Arxula adeninivorans*
Aadh2p: alcohol dehydrogenase 2 protein from *Arxula adeninivorans*
ScADH: alcohol dehydrogenase from *Saccharomyces cerevisiae*
dcw: dry cell weight
YRC: yeast rDNA integrative expression cassette
YIC: yeast integrative expression cassette
MDR: medium chain reductase/dehydrogenases family
YEPD: complex medium
YMM: yeast minimal medium
U: unit
DNSS: 3,5-dinitrosalicylic acid
ORF: open reading frame
