# Supplementary figures and images for: Aadh2p: an Arxula adeninivorans alcohol dehydrogenase involved in the first step of the 1-butanol degradation pathway
Source: Microb Cell Fact. 2016 Oct 12;15:175. doi: 10.1186/s12934-016-0573-9 (PMC5062937; doi:10.1186/s12934-016-0573-9)

## Slide 1
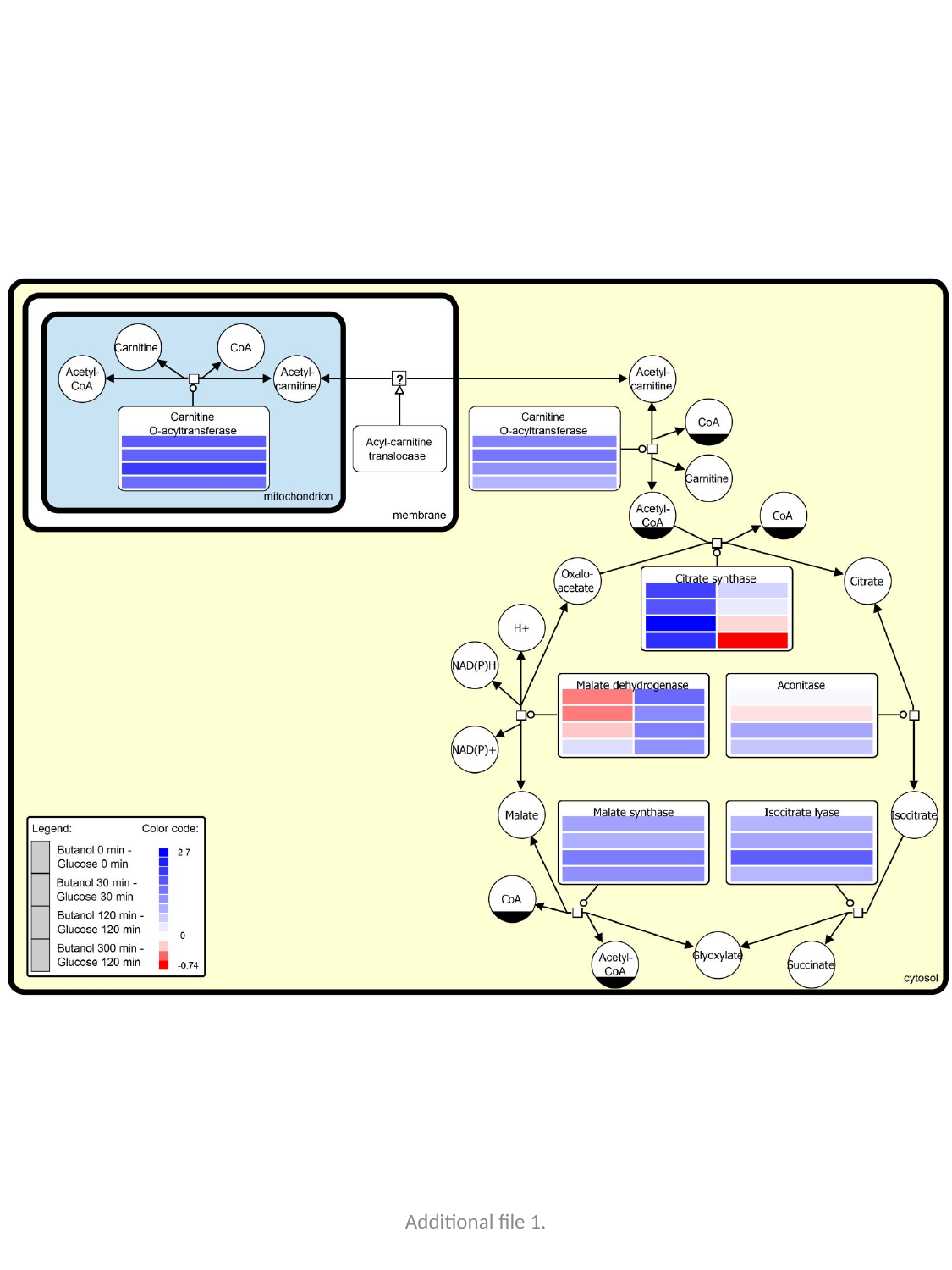

Additional file 1.

Supplement: Supplementary file 1 — 10.1186/s12934-016-0573-9 Key compounds of the glyoxylate cycle—microarray studies. The SBGN style metabolic network depicts reversible (double headed arrow) and irreversible (single headed arrow) reactions catalyzed by the corresponding enzymes (rectangular square). Enzymes are enriched with color-coded fold change values of time resolved expression data of the respective genes. The colors represent upregulation (blue) and downregulation (red) of genes in cells shifted to medium containing 1-butanol as the carbon source compared to cells grown with glucose. Metabolites or enzymes occurring multiple times in the metabolic network are decorated with a clone marker (e.g. CoA) (produced using VANTED [2, 3]). [file 12934_2016_573_MOESM1_ESM.pptx]

## Slide 1
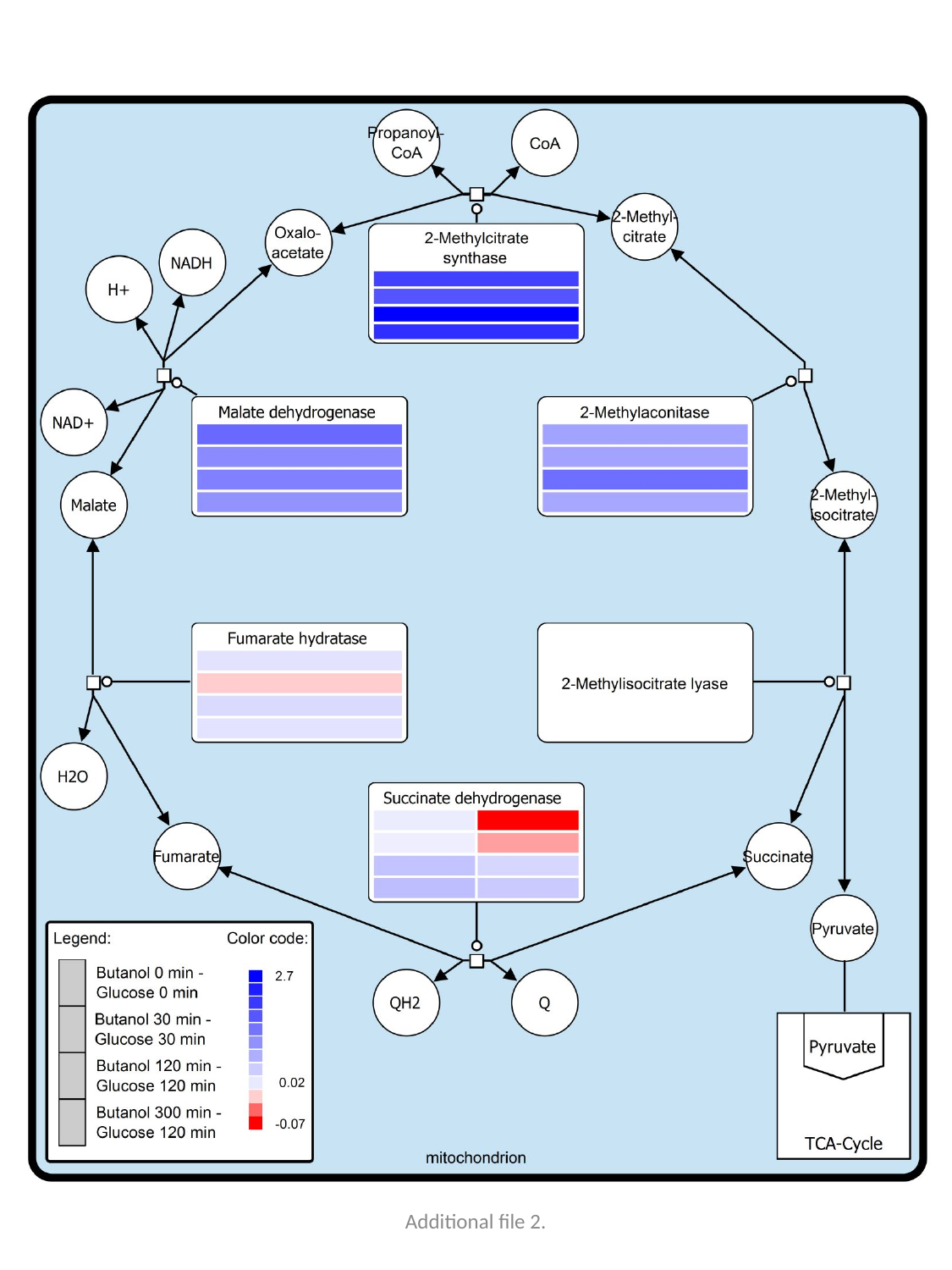

Additional file 2.

Supplement: Supplementary file 2 — 10.1186/s12934-016-0573-9 Key compounds of the methyl citrate cycle—microarray studies. The SBGN style metabolic network depicts reversible (double headed arrow) and irreversible (single headed arrow) reactions catalyzed by the corresponding enzymes (rectangular square). Enzymes are enriched with colour-coded fold change values of time resolved expression data of the respective genes. The colours represent upregulation (blue) and downregulation (red) of genes in cells shifted to medium containing 1-butanol as the carbon source compared to cells grown with glucose (produced using VANTED [2, 3]). [file 12934_2016_573_MOESM2_ESM.pptx]

## Slide 1
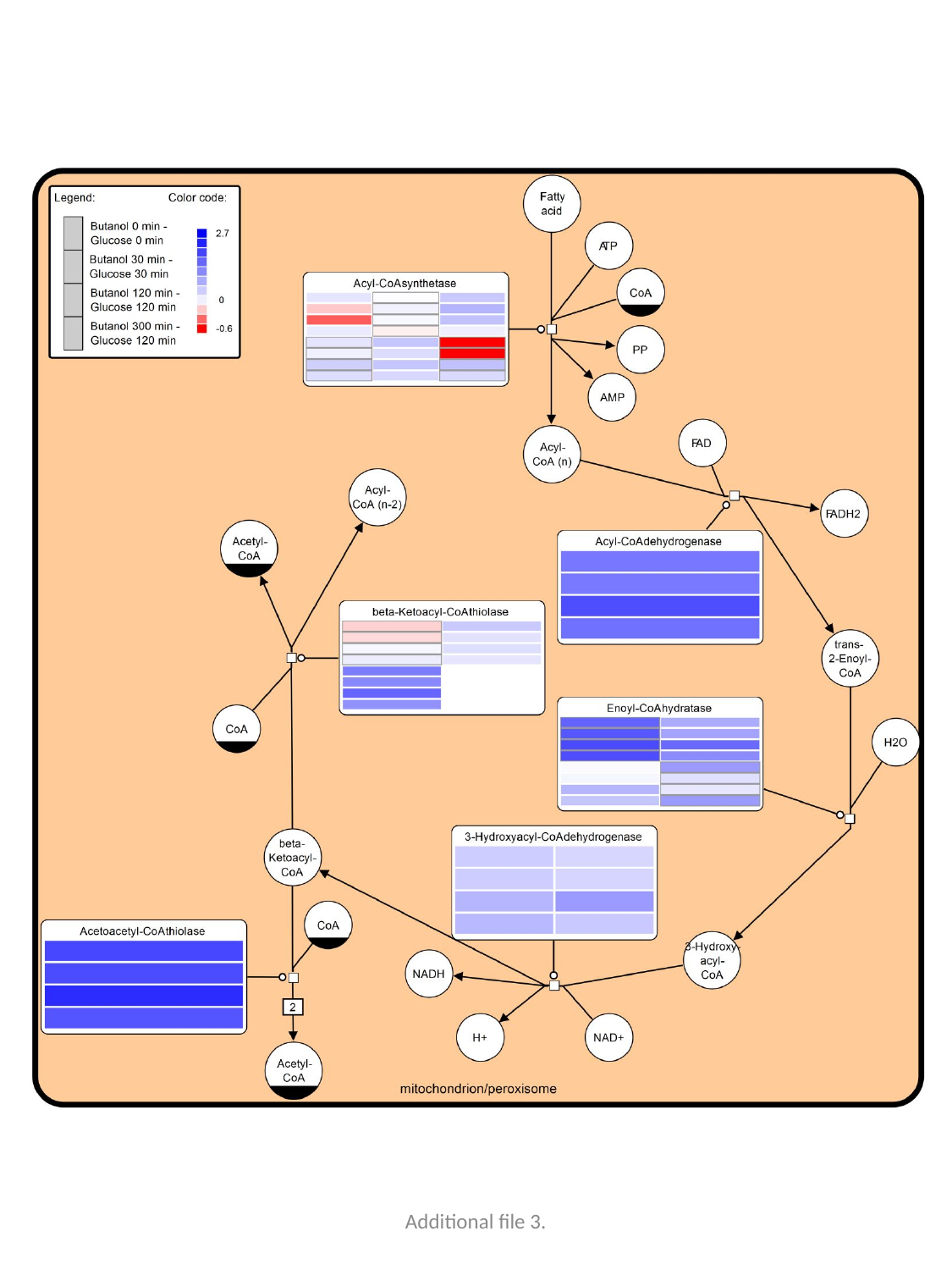

Additional file 3.

Supplement: Supplementary file 3 — 10.1186/s12934-016-0573-9 Key compounds of the ß-oxidation - microarray studies. The SBGN style metabolic network depicts reversible (double headed arrow) and irreversible (single headed arrow) reactions catalyzed by the corresponding enzymes (rectangular square). Enzymes are enriched with color-coded fold change values of time resolved expression data of the respective genes. The colors represent upregulation (blue) and downregulation (red) of genes in cells shifted to medium containing 1-butanol as the carbon source compared to cells grown with glucose. Metabolites or enzymes occurring multiple times in the metabolic network are decorated with a clone marker (e.g. CoA) (produced using VANTED [2, 3]). [file 12934_2016_573_MOESM3_ESM.pptx]
